# α-Synuclein Heterocomplexes with β-Amyloid Are Increased in Red Blood Cells of Parkinson’s Disease Patients and Correlate with Disease Severity

**DOI:** 10.3389/fnmol.2018.00053

**Published:** 2018-02-22

**Authors:** Simona Daniele, Daniela Frosini, Deborah Pietrobono, Lucia Petrozzi, Annalisa Lo Gerfo, Filippo Baldacci, Jonathan Fusi, Chiara Giacomelli, Gabriele Siciliano, Maria Letizia Trincavelli, Ferdinando Franzoni, Roberto Ceravolo, Claudia Martini, Ubaldo Bonuccelli

**Affiliations:** ^1^Department of Pharmacy, University of Pisa, Pisa, Italy; ^2^Department of Clinical and Experimental Medicine, University of Pisa, Pisa, Italy

**Keywords:** Parkinson’s disease, neurodegenerative disorders, α-synuclein, β-amyloid, tau, α-synuclein heterocomplexes

## Abstract

Neurodegenerative disorders (NDs) are characterized by abnormal accumulation/misfolding of specific proteins, primarily α-synuclein (α-syn), β-amyloid_1–42_ (Aβ_1–42_) and tau, in both brain and peripheral tissues. In addition to oligomers, the role of the interactions of α-syn with Aβ or tau has gradually emerged. Nevertheless, despite intensive research, NDs have no accepted peripheral markers for biochemical diagnosis. In this respect, Red Blood Cells (RBCs) are emerging as a valid peripheral model for the study of aging-related pathologies. Herein, a small cohort (*N* = 28) of patients affected by Parkinson’s disease (PD) and age-matched controls were enrolled to detect the content of α-syn (total and oligomeric), Aβ_1–42_ and tau (total and phosphorylated) in RBCs. Moreover, the presence of α-syn association with tau and Aβ_1–42_ was explored by co-immunoprecipitation/western blotting in the same cells, and quantitatively confirmed by immunoenzymatic assays. For the first time, PD patients were demonstrated to exhibit α-syn heterocomplexes with Aβ_1–42_ and tau in peripheral tissues; interestingly, α-syn-Aβ_1–42_ concentrations were increased in PD subjects with respect to healthy controls (HC), and directly correlated with disease severity and motor deficits. Moreover, total-α-syn levels were decreased in PD subjects and inversely related to their motor deficits. Finally, an increase of oligomeric-α-syn and phosphorylated-tau was observed in RBCs of the enrolled patients. The combination of three parameters (total-α-syn, phosphorylated-tau and α-syn-Aβ_1–42_ concentrations) provided the best fitting predictive index for discriminating PD patients from controls. Nevertheless further investigations should be required, overall, these data suggest α-syn hetero-aggregates in RBCs as a putative tool for the diagnosis of PD.

## Introduction

Parkinson’s disease (PD) represents the second most common neurodegenerative disorder (ND), with a prevalence of 1% in the population older than 60 years (Andersen et al., 2017). To date, no disease modifying treatment has been established, and the continuous pathology progression results in increasing patients’ disability (Andersen et al., 2017).

The pathologic abnormalities associated with PD are primary caused by misfolding, aggregation and brain deposition of α-synuclein (α-syn), which constitute the major components of Lewy bodies (LB) and Lewy neuritis (Spillantini et al., 1997; Dickson, 2012; Miraglia et al., 2015).

Therefore, PD belongs to a group of NDs called “synucleinopathies”, which includes dementia with Lewy bodies (DLB) and multiple system atrophy (Irizarry et al., 1998; Spillantini et al., 1998).

PD diagnosis is based on clinical examination (Hughes et al., 1992) and on patients’ response to dopaminergic drugs and can be supported by imaging techniques (Politis, 2014). Ultimate diagnosis is achieved only post-mortem, by examining *substantia nigra* damaging and the presence of LB/Lewy neuritis (Dickson, 2012; Rajput and Rajput, 2014). For these reasons, reliable biomarkers for PD diagnosis and prognosis are urgently needed. Although many efforts have been made in this direction, there is still not a specific accepted biomarker for early PD diagnosis and monitoring of disease progression (Miller and O’Callaghan, 2015).

The process of α-syn accumulation and aggregation has been demonstrated to start even decades before the onset of clinical disease symptoms (Compta et al., 2015; Miraglia et al., 2015) and to reach PD cerebrospinal and peripheral fluids (Tokuda et al., 2010). Therefore, great attempts have been devoted to exploiting substantial biological changes and putative biomarkers in tissue other than the brain. In this respect, cerebrospinal fluid (CSF) has resulted the most explored fluid because it is considered to reproduce brain pathological processes, such as synaptic/axonal degeneration (Giacomelli et al., 2017). Several studies have shown that in primary synucleinopathies, such as PD and dementia with LB, CSF of patients presented decreased levels of total α-syn and increased concentrations of its oligomeric form (Tokuda et al., 2010; Mollenhauer et al., 2013; Parnetti et al., 2014a,b; Compta et al., 2015; Giacomelli et al., 2017).

Crucial disadvantages have limited the clinical diagnostic and prognostic use of CSF biomarkers, highlighting the opportunity to look at peripheral biomarkers that could correlate with brain lesions and disease progression (Giacomelli et al., 2017). In this respect, blood has emerged as a source of neurodegeneration biomarkers because of their availability, lower cost and time effectiveness with respect to CSF. Indeed, pathological alterations in blood proteins have been suggested to reflect the changes in CSF due to simple diffusion or barrier impairment that characterizes neurodegeneration (Reiber, 2003; Giacomelli et al., 2017). In particular, α-syn in blood is mainly contained in red blood cells (RBCs; Barbour et al., 2008), which are particularly sensitive to the accumulation of misfolded proteins (Eisele et al., 2010; Pandey and Rizvi, 2010; Wang et al., 2015). For example, recently, Papagiannakis et al. (2017) have reported an increase of dimeric α-syn levels in erythrocyte membranes obtained from PD patients. Other studies have shown that the ratio oligomeric/total α-syn in RBCs is higher in PD patients than in control subjects (Abd-Elhadi et al., 2015; Wang et al., 2015; Zhao et al., 2016). Nevertheless, no correlation between RBC α-syn and disease duration, age, or motor scale score in PD patients has been evidenced (Wang et al., 2015), thus pointing out the need of further studies.

Actually, retrospective studies have highlighted that α-syn level alone is not sufficient to distinguish healthy subjects from patients with synucleinopathies or other NDs, suggesting that other predictable biomarkers are needed (Gao et al., 2015). In this respect, a mixed pattern of protein aggregates in NDs has been evidenced (Baldacci et al., 2016; Giacomelli et al., 2017). For instance, besides α-syn depositions, decreased levels of β-amyloid_1–42_ (Aβ_1–42_) and increased microtubule-associated protein tau concentrations have been shown to correlate with PD cognitive decline, thus proposing Aβ_1–42_ and tau as prognostic markers for cognitive deficits in PD (Sengupta et al., 2015). Similarly, the pathophysiologic connection between these proteins has been demonstrated in CSF of PD subjects, too (Alves et al., 2010; Montine et al., 2010).

Interestingly, besides the “clinical overlapping”, α-syn has been shown to physically interact with tau (Sengupta et al., 2015; Andersen et al., 2017) or Aβ_1–42_ (Parnetti et al., 2013; Andersen et al., 2017) and to induce the formation of hybrid oligomers (“heteroaggregates”) in PD patients’ brains. In this context, we recently demonstrated that α-syn forms heteroaggregates with Aβ_1–42_ or tau in RBCs of healthy subjects, too (Daniele et al., 2017); in the same study, a novel home-made immunoenzymatic assay has been employed to quantify such oligomers, highlighting their accumulation with increasing age and decreasing anti-oxidant capability (Daniele et al., 2017).

Herein, a cohort of PD patients and age-matched healthy controls (HC) was enrolled to explore putative changes in α-syn-tau and α-syn-Aβ_1–42_ levels between the two groups. Such reliable measurements will be not only valuable for the discovery of ideal blood biomarkers but also for a better understanding of PD pathophysiological process. Moreover, α-syn (total and oligomeric), tau (total and phosphorylated tau), and Aβ_1–42_ species were measured in the same subjects, in order to investigate the usefulness of combining different biomarkers reflecting diverse pathogenic pathways.

## Materials and Methods

### Study Population and Setting of the Study

Twenty-eight patients suffering from PD were recruited from the Department of Neurology of the Department of Medicine of the University of Pisa. In parallel, 45 age- and sex-matched HC were studied (Table 1). All subjects were free of cardiovascular disease or other major medical disorders, as assessed by clinical history and physical examination.

**Table 1:** Descriptive analysis of Parkinson’s disease (PD) patients and healthy controls (HC).

	PD	PD *de novo*	PD under therapy	HC
*N*	28	14	14	45
Age (years)	67.0 ± 9.3	64.7 ± 10.6	69.4 ± 7.5	61.9 ± 7.8
Gender	12 M/16 F	4 M/10 F	8 M/6 F	18 M/27 F
UPDRS III	22.1 ± 10.3	17.7 ± 8.0	26.1 ± 10.9*	
MMSE	28.9 ± 1.8	29.3 ± 1.2	28.5 ± 2.2	
H&Y	1.9 ± 0.7	1.5 ± 0.5	2.2 ± 0.7	
Age of onset	64.3 ± 9.4	63.4 ± 10.9	65.1 ± 8.0	
Disease duration (years)	2.8 ± 2.9			
LED (mg/day)			473 ± 268	

The data are the mean ± SD. Differences between groups—HC group, PD de novo subgroup, and PD under treatment subgroup—were evaluated by a non-parametric analysis (Kruskal Wallis): *P < 0.05 vs. PD de novo.

All patients fulfilled the diagnostic criteria for idiopathic PD (Hughes et al., 1992); atypical parkinsonian disorders, such as multiple system atrophy, progressive supranuclear palsy and corticobasal degeneration, and other neurological or major medical conditions were considered as exclusion criteria. Information about disease duration from symptoms onset and therapy were collected. Fourteen patients were newly diagnosed and drug naïve, 14 were assuming dopaminergic treatment; in these subjects, motor assessment were performed in ON state. Full clinical assessment was performed and the degree of motor impairment was scored by the Hoehn and Yahr (H&Y) rating scales (Hoehn and Yahr, 1967) and the motor (III) part of the Unified Parkinson’s Disease Rating Scale (UPDRSIII; Fahn and Elton, 1987). Global cognitive function was evaluated by Mini Mental State Examination (MMSE; Folstein et al., 1975).

This study was carried out in accordance with the recommendations of Declaration of Helsinki and Great North West Area of Tuscany guidelines, with written informed consent from all subjects. All subjects gave written informed consent in accordance with the Declaration of Helsinki. The protocol was approved by the Great North West Area of Tuscany (271/2014 to FF and 152/2016 to RC).

### RBC Collection

Whole blood was collected from patients and healthy volunteers into a tube containing EDTA as an anticoagulant. RBCs were separated from plasma by a centrifugation at 200× *g* at 4°C for 10 min (Daniele et al., 2017). The RBC pellet was centrifuged at 1000 × *g* for 10 min and washed three times with PBS. RBC pellet was frozen at −20°C until use. Protein content was determined by the Bradford method. For immunoenzymatic assays, RBC were suspended in 2 mM SDS to a final concentration of 40 mg of total proteins in 100 μl.

### Co-immunoprecipitation–Western Blotting

To confirm α-syn association with tau or Aβ_1–42_, a co-immunoprecipitation assay was employed (Daniele et al., 2017). Briefly, 1 mg of RBC lysates was resuspended in buffer and was probed o.n. under constant rotation with an anti-α-syn antibody (5 μg/sample), and then immunoprecipitated with protein A-Sepharose. After extensive washing, the immunocomplexes were resuspended in Laemmli solution, resolved by SDS-PAGE and probed o.n. with primary antibodies to α-syn (input), tau (H-150 SC-5587, Santa Cruz Biotechnology) or Aβ_1–42_ (β-amyloid H-43 SC-9129, Santa Cruz Biotechnology). The primary antibodies were detected using peroxidase-conjugated secondary antibodies. The western blot experiments were only used to investigate preliminary the presence of α -syn heterocomplexes in RBCs of PD patients, but were not used as quantitative data and thus not included in any statistical analyses.

### Immunoassay Methods for Total α-Synuclein

Total α-syn was identified in RBCs following literature’s protocols (Daniele et al., 2017). Briefly, wells were pre-coated overnight at 4°C with a full length antibody to α-syn (sc-10717, Santa Cruz Biotechnology), and then bovine serum albumin (BSA) was used to block non-specific sites for 1 h at 37°C (Daniele et al., 2017). RBCs (0.150 mg/100 μl) were captured on wells for 2 h at 25°C. Samples were then probed with a mouse monoclonal antibody (mAb) to α-syn (Santa Cruz, sc-12767), and subsequently with an anti-mouse-HRP antibody. The wells were then washed four times with PBS-T (phosphate buffered saline containing 0.01% Tween 20), the enzyme substrate 3,3′,5,5′-tetramethylbenzidine (TMB; Thermo Scientific) was added to the wells and the color was allowed to develop for 30 min at room temperature. Absorbance values at 450 nm (Daniele et al., 2017).

### Preparation of Aged Solutions of α-syn and of the α-syn Biotinylated Antibody

Recombinant α-syn were incubated in tubes at 37°C for 4 days in an Eppendorf Thermomixer with continuous mixing (1000 rpm), as reported previously (El-Agnaf et al., 2006; Daniele et al., 2017).

To prepare the α-syn biotinylated antibody, Sulfo-NHS-LC-Biotin (Pierce, Rockford, IL, USA; 200 mg) was reacted with the 211 mouse mAb (Santa Cruz Biotechnology, Santa Cruz, CA, USA; Daniele et al., 2017). To eliminate uncoupled biotin, the mixture was desalted on Bio-Spin-6 columns (BIO-RAD, UK).

### Detection of Oligomeric α-syn

Oligomeric α-syn levels in RBCs were measured using an immunoenzymatic assay, as previously described (El-Agnaf et al., 2006; Daniele et al., 2017). The plate was pre-coated overnight at room temperature with the mouse monoclonal α-syn 211 antibody (Santa Cruz, sc-12767). RBCs (0.04 mg/100 μl) were added to each well for 2 h. α-syn oligomers were identified using a specific α-syn biotinylated antibody (El-Agnaf et al., 2006; Daniele et al., 2017). Streptavidin-horseradish peroxidase conjugate antibody (1:1000, GE Healthcare) was used for antigen detection. After three washes with PBS-T, 100 μl of TMB were added in each well, as reported above.

### Detection of Total Aβ_1–42_

Aβ_1–42_ levels in blood samples were measured using an immuno-enzymatic assay, as described previously (Pesini et al., 2012; Daniele et al., 2017). Wells were pre-coated overnight at 4°C with a specific antibody to Aβ_1–42_ (Santa Cruz, sc-9129). After extensive washing with PBS-T, non-specific sites were blocked with 1% BSA, and wells were incubated with RBCs (0.2 mg/100 μl) at 25°C for 1 h. The wells were washed with PBS-T, and samples were probed using a specific antibody to Aβ_1–42_ (sc-5399, Santa Cruz Biotechnology). The standard curve was created using recombinant human Aβ_1–42_ solutions at eight different concentrations (Pesini et al., 2012; Daniele et al., 2017).

### Detection of Total Tau

Tau levels in blood samples were measured using an immuno-enzymatic assay, as described previously (Daniele et al., 2017). The plate was pre-coated overnight at 4°C with a specific antibody to tau (Santa Cruz, sc-32274), and 1% BSA was used to block non-specific sites. RBCs (0.5 mg/100 μl) were loaded to each well and incubated at 25°C for 1 h. After several washing with PBS-T, samples were detected using a tau antibody (sc-5587, Santa Cruz Biotechnology). The standard curve was constructed using recombinant human tau solutions at eight different concentrations (Daniele et al., 2017).

### Immunoassay Detection of α-syn-Aβ_1–42_ Heterocomplexes

For the quantification of α-syn-Aβ_1–42_ interactions, was used a “home-made” method employing a “sandwich” immunoenzymatic assay (Daniele et al., 2014, 2017; Zappelli et al., 2014), as follows. Eight different dilutions of α-syn-Aβ_1–42_ were prepared; following capturing on wells pre-coated with a specific antibody to Aβ_1–42_ (β-amyloid H-43 antibody, 1:100, sc-9129, Santa Cruz Biotechnology; Daniele et al., 2017) in poli-L-ornithine/NaHCO_3_, pH 9.6. After two washes with PBS-T, RBCs (40 mg/sample in 2 mM SDS) were loaded to each well and incubated at 25°C for 2 h. One percent BSA was added for 30 min at 37°C to block non-specific sites. To detect α-syn bound to Aβ_1–42_, samples were probed for 2 h at 37°C with a specific antibody to α-syn (sc-12767, Santa Cruz Biotechnology), and subsequently with the appropriate HRP-conjugated antibody. After 1.5 h, the wells were washed twice with PBS-T, and 100 μl of TMB were added to each well. Absorbance was measured at 450 nm. Relative concentrations of α-syn-Aβ_1–42_ complexes were calculated according to the standard curve obtained in each microplate. The assays of blood plasma were all carried out in duplicate. Blood samples from patients and healthy subjects were analyzed together in batch runs. For some subjects, multiple assays were performed on diluted RBCs from a single subject to confirm that low or high concentrations were in the linear range of the assay. All measurements were repeated twice and the average value was determined (Daniele et al., 2017).

### Immunoassay Detection of α-syn-tau Heterocomplexes

For the quantification of α-syn-tau interactions, was used a similar “home-made” method (see the precedent paragraph; Daniele et al., 2017). The wells were pre-coated overnight at room temperature with anti-α-syn antibody (1:100, sc-7012, Santa Cruz Biotechnology) in poli-L-ornithine/NaHCO_3_, pH 9.6. After two washes with PBS-T, RBCs (80 mg/sample in 2 mM SDS) were loaded to each well and incubated at 25°C for 2 h. The wells were washed twice, and non-specific sites were blocked with 1% BSA for 30 min at 37°C. To detect α-syn bound to tau, samples were probed for 2 h 37°C with a specific antibody to tau (sc-5587, Santa Cruz Biotechnology), and subsequently with the appropriate HRP-conjugated antibody. After 1.5 h, the wells were washed twice with PBS-T, and 100 μl of TMB were added to each well. Absorbance was measured at 450 nm. Relative concentrations of α-syn/tau complexes were calculated according to the standard curve obtained in each microplate (Daniele et al., 2017).

### Detection of Phosphorylated Tau

Phospho tau (p-tau) levels in blood samples were measured using a “home-made” immuno-enzymatic assay (Hu et al., 2002). The plate was pre-coated overnight at 25°C with a specific antibody to tau (Santa Cruz, sc-32274) in poli-L-ornithine/NaHCO_3_, pH 9.6. After extensive washing with PBS-T, 1% BSA was used to block non-specific sites. RBCs were loaded to each well and incubated at 25°C for 2 h. To detect phospho tau, samples were probed for 1.5 h 37°C with a specific antibody to p-tau (70R-32555, Santa Cruz Biotechnology), and subsequently with the appropriate HRP-conjugated antibody. After 1.5 h, the wells were washed twice with PBS-T, and 100 μl of TMB were added to each well. Absorbance was measured at 450 nm. Relative concentrations of p-tau were calculated according to the standard curve obtained in each microplate. P-tau standards were obtained from Fujirebio (Phospho-Tau Calibrator-RVC pack, Innotest Phospho-Tau (181P)). The assays of blood plasma were all carried out in duplicate.

### Statistical Analysis

Data are presented as mean value ± SD (*N* = 3). The results are expressed as ng of the measured protein/mg of the total protein content in the RBC suspension.

The population included in this study presented a normal distribution for age. When only two groups were present, the non-parametric Mean-Whitney test was used (HC subjects vs. PD patients). Differences between groups—HC group, PD *de novo* subgroup, and PD under treatment subgroup—were evaluated by a non-parametric analysis (Kruskal Wallis). Correlation between variables was determined by a Spearman analysis, while interactions between variables were calculated by correlation and multiple regression analyses. All statistical procedures were performed using the StatView program (Abacus Concepts, Inc., SAS Institute, Cary, NC, USA; Franzoni et al., 2005; Daniele et al., 2017). A receiver operating characteristic (ROC) curve was used to calculate the relationship between sensitivity and specificity for PD vs. HC, and the diagnostic performance of the measured proteins in RBCs (Zhao et al., 2016). ROC analyses were calculated using MedCalc, version 12.2.1.0 (MedCalc Software).

## Results

### Descriptive Analysis

Demographic and clinical data of the cohort of PD patients and HC are reported in Table 1. The mean age of patients at sampling was 67.0 years ± 9.3 (youngest 43, oldest 80), consisting of 12 males and 16 females. Controls (*N* = 45) were of 18 males and 27 females, and have a mean age of 61.9 years ± 7.8 (youngest 51, oldest 81).

PD subjects were divided into “*de novo*” when drug naïve or “under therapy”. Pharmacological treatments consisted in levodopa and/or dopamine agonists (Dago), alone or in combination with monoamine oxidase inhibitors (MAOIs). The therapeutic regimen was expressed as levodopa equivalent dose (LED; Tomlinson et al., 2010).

Age did not significantly differ between HC and PD patients, or between PD *de novo* or under therapy. HC, PD *de novo* or under therapy presented comparable sex distribution of subjects.

UPDRS III was significantly higher in PD under therapy with respect to *de novo* (*p* = 0.0295, Table 1). The PD and healthy subjects were recruited in parallel, and blood samples were processed in the same way, and assayed in parallel on the same multiwell plate for each immunoenzymatic assay.

### Immunoassay Results

#### HC vs. PD Patients

Levels of α-syn (total and oligomeric), tau (total and phosphorylated) and Aβ_1–42_ were quantified by immunoenzymatic assays, and reported in Table 2.

**Table 2:** Concentrations of total α-syn, oligomeric α-syn, Aβ_1–42_, α-syn-Aβ_1–42_, tau, p-tau and α-syn-tau in the indicate subgroups.

	Total α-syn (ng/mg protein)	Oligomeric α-syn (ng/mg protein)	Aβ_1–42_ (ng/mg protein)	α-syn-Aβ_1–42_ (ng/mg protein)	Tau (ng/mg protein)	p-tau (pg/mg protein)	α-syn-tau (ng/mg protein)
PD	11.1 ± 8.0**	8.20 ± 5.16**	11.4 ± 9.9	9.18 ± 11.67***	13.8 ± 21.8*	282 ± 105**	2.29 ± 1.74
PD *de novo*	12.5 ± 7.3*	9.90 ± 2.69***	8.53 ± 4.53	14.4 ± 14.8*	19.0 ± 26.4**	305 ± 100**	1.94 ± 1.05
PD under therapy	9.75 ± 8.63*	6.52 ± 5.65^#^	14.3 ± 12.9	3.99 ± 2.13^##^	8.55 ± 15.13	259 ± 109	2.65 ± 2.21
HC	49.1 ± 63.0 (90th-percentile HC: 32.4 ± 31.7)	5.31 ± 1.59	13.9 ± 12.4	2.90 ± 2.01	6.02 ± 3.50	195 ± 81	2.23 ± 1.66

The values are expressed as mean ± SD. Differences between groups—HC group, PD de novo subgroup, and PD under treatment subgroup—were evaluated by a non-parametric analysis (Kruskal Wallis): *P < 0.05, **P < 0.01, ***P < 0.001 vs. HC, ^#^*P* < 0.05, ^##^*P* < 0.01 vs. PD de novo.

The RBC concentrations of α-syn were comparable to those previously reported from independent authors (Barbour et al., 2008). Due to the high standard deviation of total α-syn values in the HC population, the frequency distribution of such parameter was analyzed (Supplementary Figure S1A). The distributions of values led us to select a population of HC presenting RBC α-syn concentrations of 129.46 ng/mg protein or lower (90th percentile, Supplementary Figure S1A), on which further comparison analyses were performed (see below).

**Figure 1:**
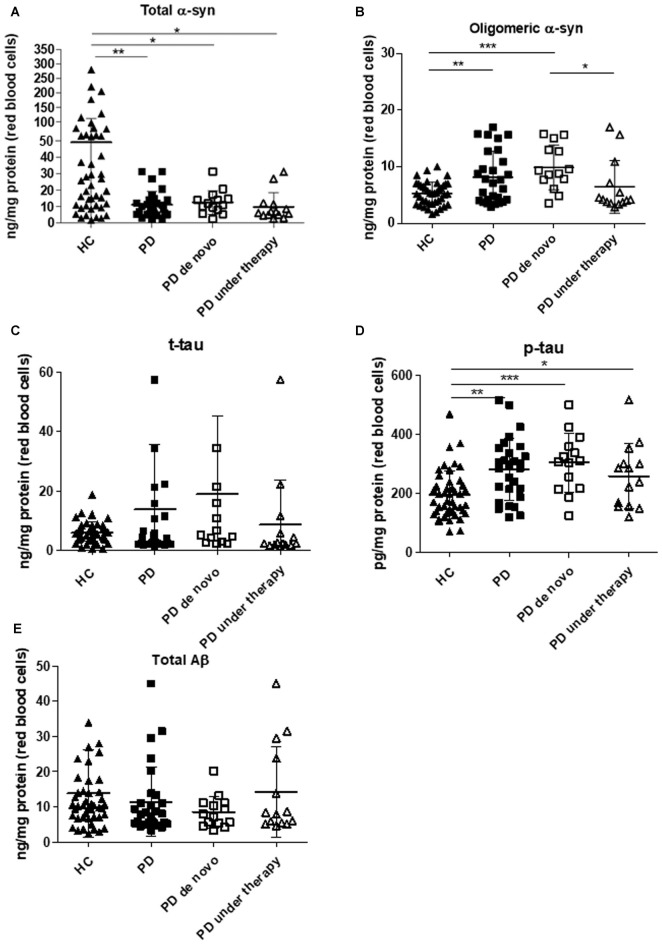
Immunoassay determinations in Red Blood Cells (RBCs) of Parkinson’s disease (PD) patients and healthy controls (HC). **(A–E)** RBC levels of total **(A)** and oligomeric **(B)** α-synuclein (α-syn), total tau **(C)**, p-tau **(D)** and Aβ_1–42_ **(E)** in the cohort of PD patients (*de novo* or under therapy) and HC (mean ± SD). Lysates obtained from RBCs were subjected to specific immunoassays, as described in the “Materials and Methods” section. Differences between groups—HC group, PD *de novo* subgroup, and PD under treatment subgroup—were evaluated by a non-parametric analysis (Kruskal Wallis): *P < 0.05, **P < 0.01, ***P < 0.001 vs. HC.

Total α-syn in RBCs showed significant lower levels in PD patients with respect to HC (Figure 1A, *P* = 0.0003). Such difference was maintained considering the 90th percentile-HC (*P* = 0.0010, Supplementary Figure S1B).

**Figure 2:**
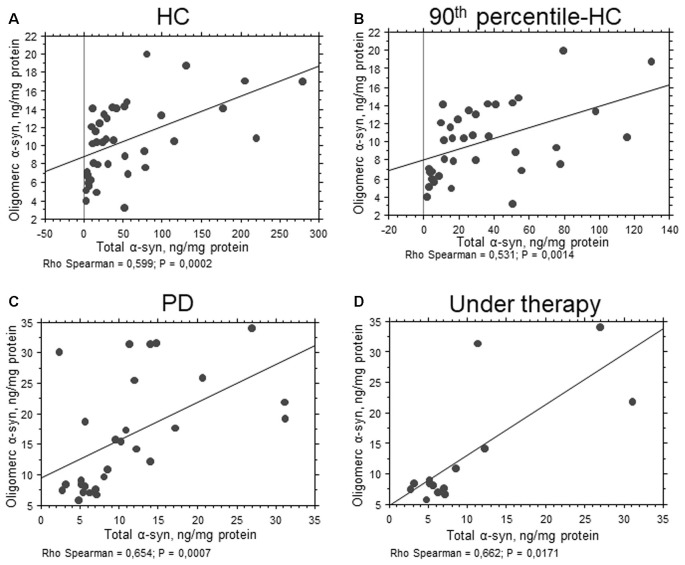
Correlation between total and oligomeric α-syn in RBCs. **(A–D)** Correlation analysis between total and oligomeric α-syn concentrations in RBCs of HC **(A)**, 90th percentile-HC **(B)**, PD **(C)** and PD under therapy **(D)**. Correlations were determined using a Spearman analysis. A regression line was shown for representative purpose.

In contrast to total α-syn, an opposite trend was found for the oligomeric form of the protein (Figure 1B, *P* = 0.0110). The calculated ratio oligomeric/total α-syn resulted significantly elevated in patients with respect to HC (*P* < 0.0001). Furthermore, a significant positive correlation between total and oligomeric α-syn concentrations in RBCs was found in the total population (*P* = 0.0010, Rho Spearman = 0.398), as well as in HC (HC: *P* = 0.0002, Rho Spearman = 0.599; 90th percentile-HC: *P* = 0.0014, Rho Spearman = 0.531, Figures 2A,B) and in the PD cohort (*P* = 0.0007, Rho Spearman = 0.654, Figure 2C).

Interestingly, the levels of phosphorylated tau (p-tau, Figure 1D, *P* = 0.0003) were significantly higher in the PD cohort. Nevertheless, neither the total protein (t-tau, Figure 1C, *P* = 0.5105) or the ratio p-tau/t-tau (*P* = 0.0713) differed between PD subjects and HC. Similarly, total Aβ_1–42_ concentrations were comparable in the two groups (Figure 1E, *P* = 0.2079), suggesting that total tau and Aβ_1–42_ are not compromised in the analyzed cohort of PD patients.

**Figure 3:**
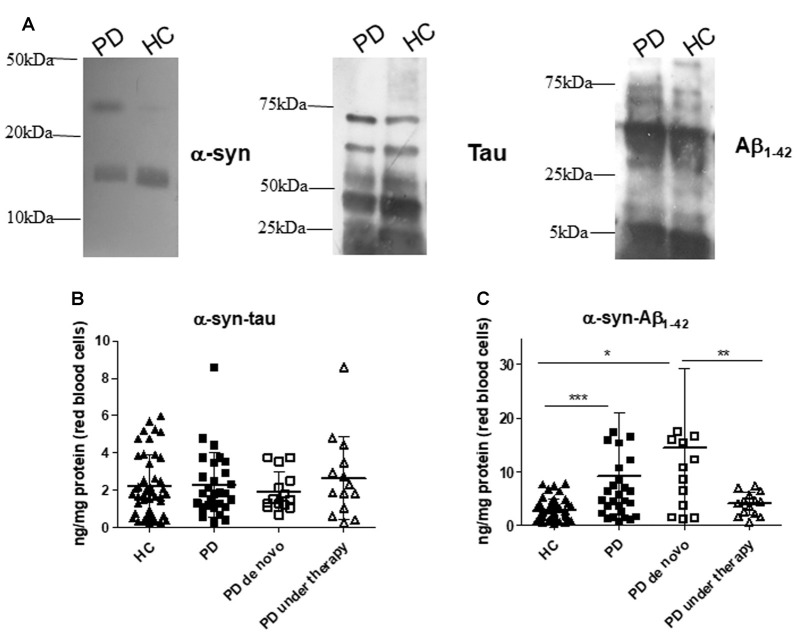
α-syn heterocomplexes with tau and Aβ_1–42_ in RBCs. **(A)** Cell lysates obtained from RBCs of HC and PD patients were immunoprecipitated with an anti-α-syn antibody, and then immunoblotted with antibody to α-syn, Aβ_1–42_ or tau. One representative Western blot is presented for each condition. **(B,C)** RBC levels of α-syn-tau **(B)** or α-syn-Aβ_1–42_ **(C)** and in the cohort of PD patients (*de novo* or under therapy) and HC (mean ± SD). Lysates obtained from RBCs were subjected to specific immunoassays, as described in the “Materials and Methods” section. Differences between groups—HC group, PD *de novo* subgroup, and PD under treatment subgroup—were evaluated by a non-parametric analysis (Kruskal Wallis): *P < 0.05, **P < 0.01, ***P < 0.001 vs. HC.

Finally, α-syn association with Aβ_1–42_ or tau were quantified in RBCs (Figure 3 and Table 2). Recently, we have demonstrated the presence of such heterocomplexes in human RBCs, and validated an immunoassay for their determination (Daniele et al., 2017). Co-immunoprecipitation analysis qualitatively showed that α-syn associated with Aβ_1–42_ and tau in RBCs from HC and PD patients (Figure 3A and Supplementary Figure S2). Such results are consistent with those obtained in RBCs (Daniele et al., 2017) and platelets (Daniele et al., 2018) of HC and confirm the existence of hybrid oligomers at peripheral level.

Quantitative immunoassays showed comparable RBC concentrations of α-syn heterocomplexes with tau (Figure 3B, *P* = 0.8738); in contrast, α-syn-Aβ_1–42_ heteroaggregate levels were found to be significantly higher in PD patients with respect to HC (Figure 3C, *P* = 0.0014).

#### HC vs. PD *de novo* or Under Therapy

Statistical analyses were repeated dividing PD patients in “*de novo*” (*N* = 14) or under therapy (*N* = 14).

As shown in Figure 1 and in Supplementary Figure S1, HC presented higher levels of total α-syn with respect to *de novo* (whole HC: *P* = 0.0150, Figure 1A; 90th percentile-HC: *P* = 0.0252, Supplementary Figure S1B) or treated (whole HC: *P* = 0.0015, Figure 1A; 90th percentile-HC: *P* = 0.0036, Supplementary Figure S1B) patients, whereas comparable concentrations were found between *de novo* and under therapy subjects (*P* = 0.1029, Figure 1A).

Significant lower levels of oligomeric α-syn were found in HC with respect to PD *de novo* only (*P* < 0.0001, Figure 1B). Additionally, a slight but significant decrease in the oligomeric protein concentrations were found in PD patients under therapy with respect to “*de novo*” subjects (*P* = 0.0203, Figure 1B). Finally, a significant positive correlation between total and oligomeric α-syn concentrations in RBCs was evidenced in patients under therapy (*P* = 0.0171, Rho Spearman = 0.662, Figure 2D).

HC presented significant lower phosphorylated tau levels with respect to PD *de novo* (*P* = 0.0004, Figure 1D) and under therapy (*P* = 0.0363, Figure 1D). Nevertheless, the two groups of PD patients (i.e., *de novo* vs. under therapy) showed comparable RBC concentrations of p-tau (*P* = 0.1611, Figure 1D).

*De novo* and under therapy patients showed comparable RBC concentrations of total tau (*P* = 0.628 Figure 1C), Aβ_1–42_ (*P* = 0.42142, Figure 1C) and α-syn/tau (*P* = 0.5053, Figure 3B). In contrast, α-syn-Aβ_1–42_ (*P* = 0.0155, Figure 3C) concentrations were found to be significantly lower in PD patients under therapy with respect to “*de novo*” subjects. As a result, HC presented significant lower α-syn-Aβ_1–42_ levels in PD *de novo* only (HC vs. *de novo*: *P* = 0.0009; HC vs. under therapy: *P* = 0.0888, Figure 3C). Overall, these results suggest that therapy can affect α-syn association with Aβ_1–42_.

### Association with Gender and Age

The RBC levels of all the measured species did not show any association with gender in the total population, as well as in the PD or in the HC group.

**Figure 4:**
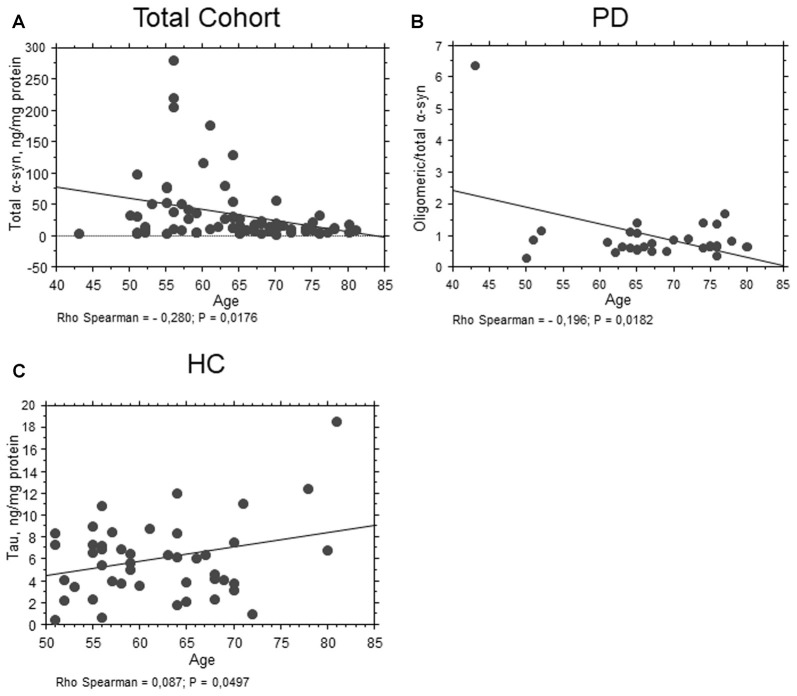
Correlation between RBC proteins and age. **(A)** Correlation analysis between age and total α-syn concentrations in RBCs of the total population. **(B)** Correlation analysis between oligomeric/total α-syn concentrations of PD patients and age. **(C)** Correlation analysis between age and total tau concentrations in RBCs of HC. Correlations were determined using a Spearman analysis. A regression line was shown for representative purpose.

As concern age, total α-syn levels showed an inverse correlation with age in the whole population (*P* = 0.0176, Rho Spearman = −0.280, Figure 4A). Moreover, an inverse correlation between the ratio oligomeric/total α-syn and age (*P* = 0.0182, Rho Spearman = −0.196, Figure 4B) was found in the PD cohort. Conversely, a positive correlation between total tau and age was found in control subjects (*P* = 0.0497, Rho Spearman = 0.087, Figure 4C).

### Correlations with Clinical Scales in PD Patients

**Figure 5:**
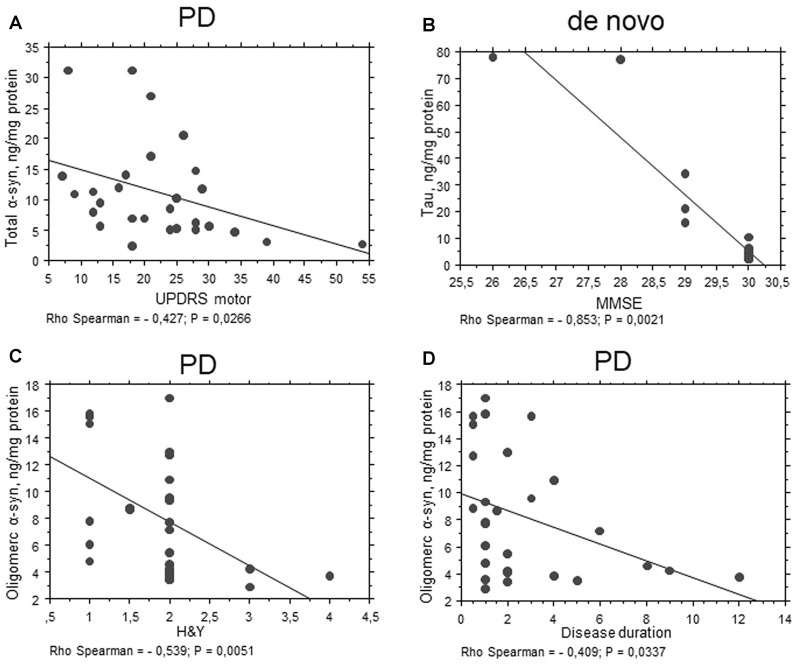
Correlation between RBC proteins and clinical scales in PD patients. **(A–D)** Correlation analysis between Unified Parkinson’s Disease Rating Scale (UPDRS) motor **(A)**, mini mental state examination (MMSE) **(B)**, H&Y **(C)** scores or disease duration **(D)** and the levels of the indicated proteins in RBCs of PD patients (total cohort or *de novo*). Correlations were determined using a Spearman analysis. A regression line was shown for representative purpose.

An inverse correlation between total α-syn and the UPDRS motor score was found (*P* = 0.0266, Rho Spearman = −0.427, Figure 5A) in the total PD cohort. These data are consistent with a decrease in α-syn concentrations evidenced in PD patients with respect to HC.

Interestingly, total tau concentrations were inversely related to MMSE in the “*de novo*” cohort of PD patients (*P* = 0.0021, Rho Spearman = −0.853, Figure 5B). These results suggest an involvement of total tau in RBCs can be related to cognitive decline.

**Figure 6:**
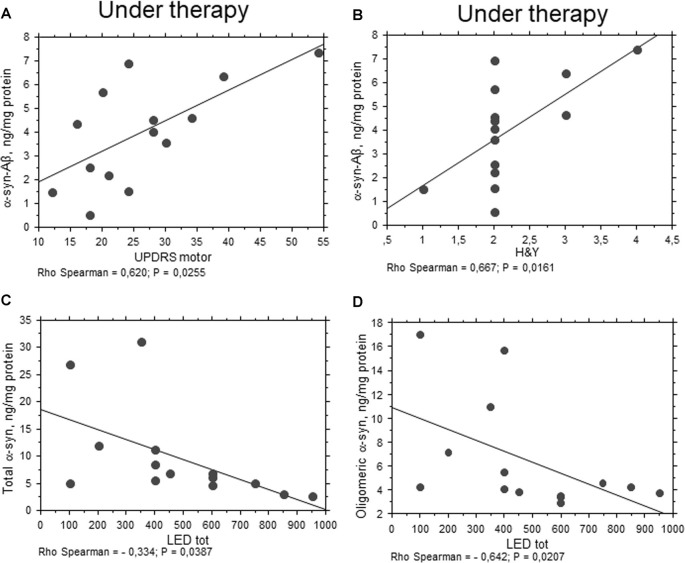
Correlation between RBC proteins and pharmacological treatment in PD patients. **(A,B)** Correlation between UPDRS motor **(A)** or H&Y **(B)** scores and the levels of the indicated proteins in RBCs of PD patients under therapy. **(C,D)** Correlation analysis between the concentrations of the indicated proteins and levodopa equivalent dose (LED). Correlations were determined using a Spearman analysis. A regression line was shown for representative purpose.

Oligomeric α-syn levels in RBCs were found to be inversely related to H&Y scale (*P* = 0.0051, Rho Spearman = −0.539, Figure 5C). A similar trend was found for the UPDRS score, although without reaching statistical significance (*P* = 0.0545, Rho Spearman = −0.370). Overall, these data may suggest oligomeric α-syn levels in RBCs decline with disease progression. Consistent with this hypothesis, oligomeric α-syn concentrations inversely correlated with disease duration (*P* = 0.0337, Rho Spearman = −0.409, Figure 5D). However, it should be considered that patients with a longer disease history present a higher age and follow a therapeutic regimen (see “Discussion” section). In this respect, α-syn-Aβ_1–42_ concentrations in RBCs correlated with the UPDRS score (*P* = 0.0255, Rho Spearman = 0.620, Figure 6A), as well as with the H&Y scale (*P* = 0.0161, Rho Spearman = 0.667, Figure 6B) in patients under pharmacological treatment (see “Discussion” section).

### Correlations with Pharmacological Treatments

Any significant correlation with LEDs (i.e., LEDs) was evidenced for Aβ_1–42_, tau and α-syn heroaggregates. Consistent with the data depicted in Figure 1B, oligomeric α-syn content in RBCs negatively correlated with LEDs (*P* = 0.0207, Rho Spearman = −0.642, Figure 6D). Surprisingly, similar findings were found for the total RBC levels of α-syn (*P* = 0.0387, Rho Spearman = −0.334, Figure 6C).

### Covariate Analysis

Covariate analysis confirmed that total tau concentrations in RBCs are most related to the MMSE score in the total cohort of PD patients (*P* = 0.0059, total *P* = 0.0301) and in the *de novo* one (*P* < 0.0001, total *P* < 0.0001). Any other correlation was found for the other analyzed parameters.

### Receiver Operating Curve (ROC) Analysis

**Figure 7:**
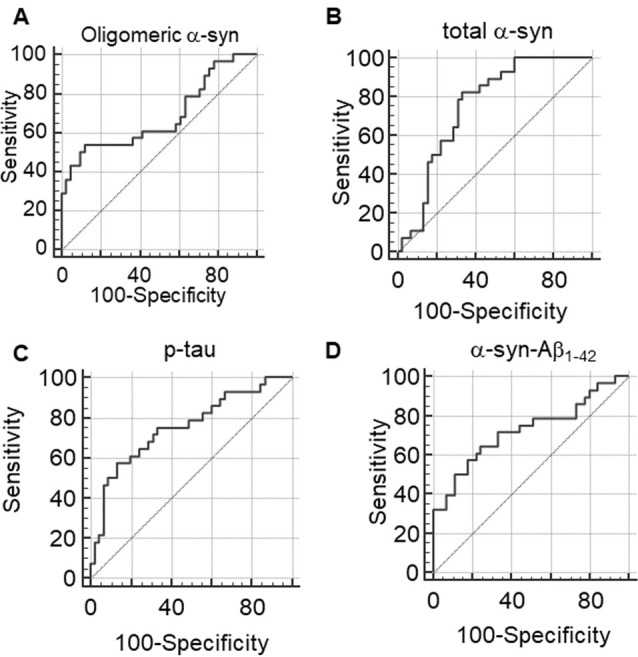
**(A–D)** Receiver Operating Characteristic (ROC) curves to evaluate the utility of RBC concentrations of oligomeric α-syn **(A)**, total α-syn **(B)**, p-tau **(C)** or α-syn-Aβ_1–42_ **(D)** in discriminating PD patients from HC.

ROC analyses were performed to evaluate the utility of measuring RBC concentrations of α-syn, tau and Aβ_1–42_ species as a means of discriminating between PD patients and HC. The area under the curve (AUC) provides an indication of predictive value, with AUC = 0.5 for a random association and AUC = 1 for perfect discrimination (Alemayehu and Zou, 2012). A modest AUC (0.681, Figure 7A) was observed for oligomeric α-syn (*P*_Area0.5_ = 0.0098, Table 3). Interesting results were obtained analysis total α-syn concentrations (sensitivity = 82.14, AUC = 0.751, *P*_Area0.5_ < 0.0001, Figure 7B and Table 3) and p-tau (AUC = 0.751, *P*_Area0.5_ < 0.0001, Figure 7C), suggesting their potential diagnostic values. Finally, a promising predictive value was observed for α-syn-Aβ_1–42_ (AUC = 0.724, *P*_Area0.5_ = 0.0007, Figure 7D and Table 3). A combination of different biomarkers in RBCs significantly predicts PD with an AUC of 0.982.

**Table 3:** Parameters of ROC analyses for RBC levels of total α-syn, oligomeric α-syn, α-syn-Aβ_1–42_ and p-tau.

ROC parameters	Total α-syn	Oligomeric α-syn	α-syn-Aβ_1–42_	p-tau
AUC	0.751	0.681	0.724	0.751
*P*_Area0.5_	*P* < 0.0001	*P* = 0.0098	*P* = 0.0007	*P* < 0.0001
Sensitivity (%)	82.14	56.57	64.29	57.14
Specificity (%)	66.67	87.80	75.56	87.67

*PArea0.5 indicates the statistical significance for Area = 0.5*.

## Discussion

In the present study, the accumulation of pathological ND-linked proteins in red blood cells of a small cohort of PD patients was evaluated as potential marker of pathology. The main conclusions of this work are as follows: (i) total α-syn was demonstrated to interact with Aβ_1–42_ and tau in RBCs of PD patients; (ii) α-syn-Aβ_1–42_ heterocomplex concentrations were elevated in PD patients, and directly correlated with disease severity and motor deficits in patients under therapy; (iii) additionally, PD patients presented decreased concentrations of total α-syn and increased levels of its oligomeric form, as well as of phosphorylated tau; and (iv) total α-syn levels were inversely related to motor deficits of PD patients. Finally, a combination of different biomarkers in RBCs significantly predicts PD diagnosis with a AUC of 0.982.

A huge amount of data has evidenced that PD hallmarks are represented by cerebral and peripheral accumulation of α-syn, even years or decades before the onset of clinical disease (Compta et al., 2015; Miraglia et al., 2015). These data support the concept that detection of accumulated/misfolded forms of abnormal proteins in peripheral biological fluids may provide a useful tool for an early diagnosis.

A mixed pattern of proteinopathies have been identified in brains of patients affected by NDs (Baldacci et al., 2016; Giacomelli et al., 2017). Moreover, in addition to homoaggregates, α-syn, tau and Aβ have been shown to interact each other or with other “pathological proteins” to form toxic heteroaggregates in both cellular models and patients’ brain (Parnetti et al., 2013; Sengupta et al., 2015; Andersen et al., 2017; Giacomelli et al., 2017). These latest findings are overcoming the concept that each neurodegenerative disease is related to the misfolding of a single specific protein (Baldacci et al., 2016). Herein, a cohort of *de novo* and under treatment patients affected by PD was enrolled to measure α-syn, tau and Aβ_1–42_ species in blood. In particular, RBCs were chosen among blood cells, based on recent findings demonstrating an accumulation of misfolded proteins in these cells and its relationship with NDs (Eisele et al., 2010; Pandey and Rizvi, 2010; Kiko et al., 2012; Brown et al., 2013; Wang et al., 2015).

In RBCs isolated from PD patients, total α-syn was significantly lower, while the oligomeric form of the protein and the ratio oligomeric/total α-syn resulted significantly higher. These data are consistent with the recent findings reported in RBCs from PD patients (Wang et al., 2015; Zhao et al., 2016; Papagiannakis et al., 2017). Several studies have assessed the levels of α-syn in *serum* or plasma of PD patients reporting controversial findings (Atik et al., 2016). In this respect, one key contributor to the conflicting results is that RBCs are a major source of α-syn, accounting for more than 99% of its blood levels, with the remainder in plasma (Barbour et al., 2008).

Total α-syn levels showed an inverse correlation with age in the whole population. Furthermore, consistent with its decreased levels in patients, an inverse correlation between total α-syn and the UPDRS motor score was found in the total PD cohort. Otherwise, in a previous article, no correlation between RBC α-syn oligomer levels and age at onset, disease duration, age, UPDRS motor scale score or progression of motor degeneration in PD patients (Wang et al., 2015) was observed.

We found that oligomeric α-syn levels in RBCs were inversely related to disease severity (i.e., H&Y score), thus suggesting that the oligomeric protein levels in RBCs may decline along with disease progression. In contrast, two independent studies have shown that CSF oligomeric α-syn levels increase in advanced PD patients with dementia (Compta et al., 2015). However, in interpreting our results, it should be considered that patients with a longer disease history generally present a higher age and follow a therapeutic regimen. In this respect, subjects under therapy presented significant lower RBC concentrations of oligomeric α-syn with respect to *de novo* patients. Moreover, oligomeric α-syn levels showed an inverse correlation with LEDs, thus supporting the hypothesis that drugs can affect the content of the protein. In this respect, dopaminergic agonists and MAO inhibitors have been demonstrated to reduce α-syn aggregation both *in vitro* (Ono et al., 2007) and in a *Drosophila* model of synucleinopathy (Yedlapudi et al., 2016). On the other hand, Levodopa administration, in particular in long-time therapies, can lead to increased oxidative stress and inflammatory response of PD patients (Dorszewska et al., 2014) even in peripheral blood cells (Dorszewska and Kozubski, 2011), possibly influencing protein accumulation in RBCs, which are particularly sensitive to oxidative stress (Pandey and Rizvi, 2011). Similarly, the MAO blocker selegiline, contrary to rasagiline, has been shown to induce a more compact α-syn structure (Kakish et al., 2015). Therefore, the different contributions of medical treatment on α-syn accumulation/aggregation in RBCs should be considered. Further experiments on a larger cohort of PD patients under therapy are ongoing in order to dissect the contribution of Levodopa, dopaminergic agonists or MAO inhibitors on synuclein misfolding.

PD patients showed an enhancement in the phosphorylated, but not total, levels of tau with respect to HC, thus supporting the hypothesis of a tau involvement in PD pathogenesis. Consistent with our data, histopathological studies have detected phosphorylated tau in neurofibrillary tangles, LB and neurites of PD and DLB cases (Arima et al., 2000; Ishizawa et al., 2003), where it colocalizes with α-syn. Conversely, decreased levels of total and/or phoshorylated tau have been found in CSF or in plasma (Sparks et al., 2012) of subjects with PD, Alzheimer’s disease (AD) and MCI compared to cognitively normal controls, with a significant positive correlation between changing levels of plasma tau and cognitive performance (Sparks et al., 2012). Besides the different NDs, such discrepancy may be due to the different blood fluid analyzed in the studies, and highlights that further investigations are needed to clarify the benefits of blood tau as a biomarker in PD and other NDs.

A positive correlation between total tau and age was found in control and in PD subjects, although without statistical significance in the latter cohort. Interestingly, total tau concentrations were directly related to cognitive deficits in PD patients in the “*de novo*” cohort. These results suggest an involvement of total tau in RBCs can be related to cognitive decline. Consistent with our data, tau aggregation/phosphorylation have been shown to increase in PD dementia (PDD) with respect to PD (Compta et al., 2009; Hall et al., 2012) and to correlate with cognitive status (Jellinger, 2007). Nevertheless, it should be noticed that our cohort of PD patients presented quite normal cognitive state. To overcome such limitation, the contribute of total and phosphorylated tau in PD with dementia will be explored in a future study.

Total levels of Aβ_1–42_ in RBCs did not differ between PD and HC. Consistent with our findings, the protein levels in CSF have been demonstrated not to differ in PD compared with controls (Mollenhauer et al., 2011). However, studies on Aβ_1–42_ contribution in PD are still in progress. Considering the striking role of Aβ_1–42_ in PD conversion to dementia (Compta et al., 2013; Alves et al., 2014) and its accumulation with age in RBCs (Kiko et al., 2012), an interesting development of our study will be to assess Aβ_1–42_ levels in PD patients with marked cognitive deficits.

Interestingly, in this study it was demonstrated for the first time that PD patients exhibited α-syn heterocomplexes with Aβ_1–42_ and tau in peripheral tissues too, as demonstrated previously in patients’ brains (Parnetti et al., 2013; Sengupta et al., 2015; Andersen et al., 2017). By the mean of a validated immunoenzymatic assay, the levels of α-syn heterocomplexes were quantified: HC presented comparable RBC concentrations of α-syn-tau with respect to PD subjects, while a highly significant enhancement of α-syn-Aβ_1–42_ species was found in pathological RBCs. No correlation with disease duration or severity was found in the total PD cohort, probably due to the small number of subjects. Nevertheless, a promising predictive value was observed for α-syn-Aβ_1–42_, with an AUC of 0.714.

Interestingly, α-syn-Aβ_1–42_ concentrations were significantly lower in PD patients under therapy with respect to “*de novo*”. It is possible that the effect of disease on α-syn-Aβ_1–42_ concentrations is overcome by the effect of age (the values decrease with age), whereas as a mere disease effect, in the cohort under pharmacological treatment, α-syn-Aβ_1–42_ concentrations in RBCs would increase with worse motor deficits and disease severity.

Herein, α-syn was demonstrated to interact with Aβ_1–42_ and tau in RBCs of PD patients; in particular, α-syn-Aβ_1–42_ concentrations were elevated in PD subjects and directly correlated with disease severity and motor deficits in patients under therapy. Moreover, PD was confirmed to be associated with decreased concentrations of total α-syn, which inversely related to motor deficits of patients. Furthermore, an enhancement of oligomeric α-syn, total and phosphorylated tau was observed, with a direct correlation between total tau concentrations and cognitive impairment.

Some limitations have to be considered in this study: the small cohort of subjects and the unavailability of CSF samples from patients, which would be necessary to correlate exactly RBCs-CSF data. Further studies on larger cohort of pathologically confirmed subjects will overcome these limitations, shedding light on the real sensitivity and specificity of α-syn heterocomplexes in RBCs.

## Author Contributions

SD, DP, CG, JF and LP conducted the experiments. DF, FB and FF recruited subjects. SD, DF, ALG and LP analyzed the data. SD, DF and FF wrote the manuscript. MLT, FF, GS, RC, UB and CM designed the study and provided overall supervision for the project. All authors contributed to the drafting and critical revision of the manuscript and have given final approval of the version to be published.

## Conflict of Interest Statement

The authors declare that the research was conducted in the absence of any commercial or financial relationships that could be construed as a potential conflict of interest.

## Abbreviations

Aβ_1–42_: β-amyloid_1–42_
α-syn: α-synuclein
AD: Alzheimer’s disease
CSF: cerebrospinal fluid
DLB: Dementia with Lewy Bodies
HC: healthy controls
LED: Levodopa equivalent dose
ND: Neurodegenerative disorder
PD: Parkinson’s disease
RBCs: Red blood cells
ROC: Receiver Operating Characteristic.
