# Supplementary material for: α-Synuclein Heterocomplexes with β-Amyloid Are Increased in Red Blood Cells of Parkinson’s Disease Patients and Correlate with Disease Severity
Source: Front Mol Neurosci. 2018 Feb 22;11:53. doi: 10.3389/fnmol.2018.00053 (PMC5827358; doi:10.3389/fnmol.2018.00053)
Supplement: Supplementary file 1 [file Image_1.pdf]

Supplementary Material

α-synuclein heterocomplexes with β-amyloid are increased in red blood cells of Parkinson's Disease patients and correlate with disease severity

Simona Daniele[1#*], Daniela Frosini[2#], Deborah Pietrobono[1], Lucia Petrozzi[2], Annalisa Lo Gerfo[2], Filippo Baldacci[2], Jonathan Fusi[2], Chiara Giacomelli[1], Gabriele Siciliano[2], Maria Letizia Trincavelli[1*], Ferdinando Franzoni[2], Roberto Ceravolo[2*], Claudia Martini[1], Ubaldo Bonuccelli[2].

[1]Department of Pharmacy, University of Pisa, 56126 Pisa, Italy.

[2]Department of Clinical and Experimental Medicine, University of Pisa, 56120 Pisa, Italy.

# These authors equally contributed to this work.

*** Corresponding authors:** Department of Pharmacy, University of Pisa, Via Bonanno 6, 56126 Pisa, Italy, and Department of Clinical and Experimental Medicine, University of Pisa, Via Savi 10, 56126, Pisa, Italy. E-mail addresses: maria.trincavelli@unipi.it; simona.daniele@unipi.it; roberto.ceravolo@unipi.it.

Supplementary Figure 1

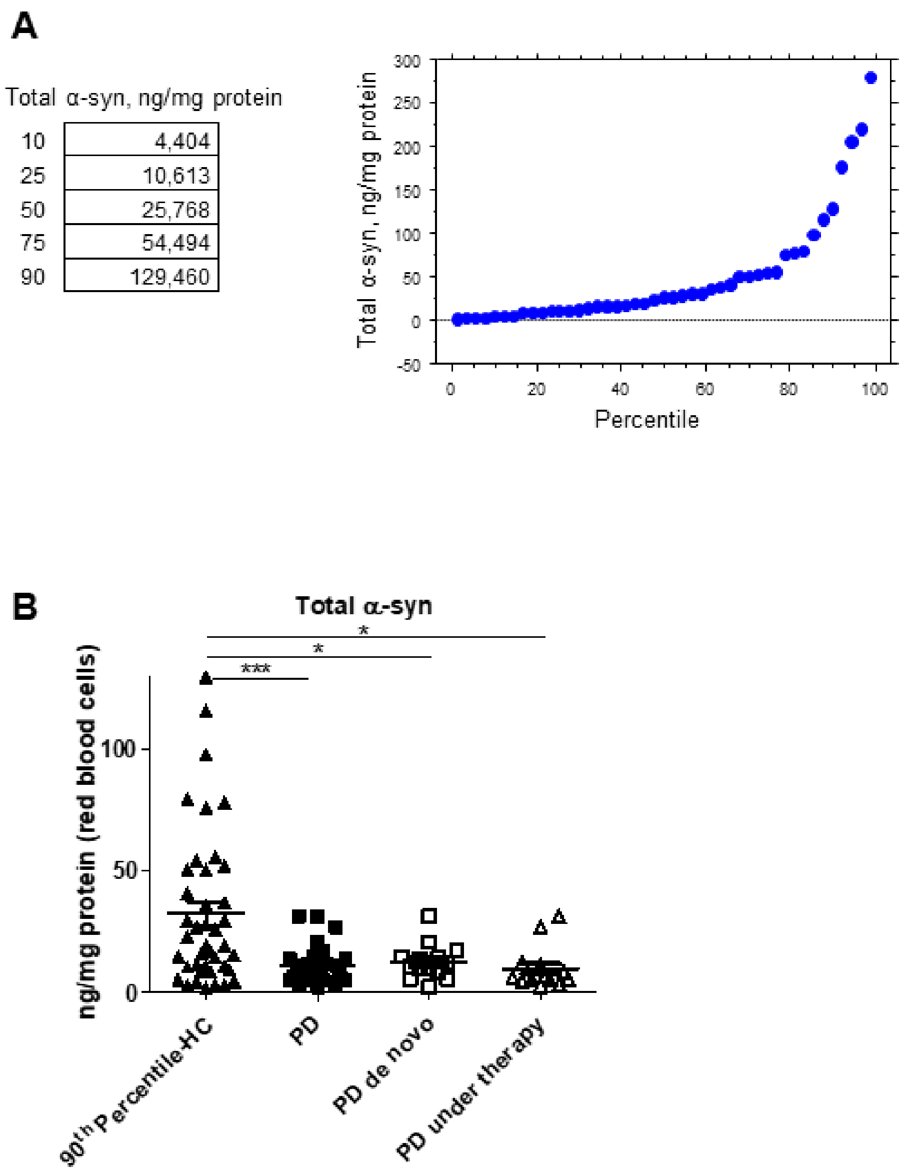

Supplementary Figure 1. (**A**) Percentile analyses of RBC levels of total α-syn in the HC cohort (**B**) RBC levels of total α-syn in the cohort of PD patients (de novo or under therapy) and 90th percentile-HC (mean ± SD). Differences between groups – HC group, PD de novo subgroup, and PD under treatment subgroup – were evaluated by a non-parametric analysis (Kruskal Wallis): *P<0.05, ***P<0.001 versus 90th percentile-HC.

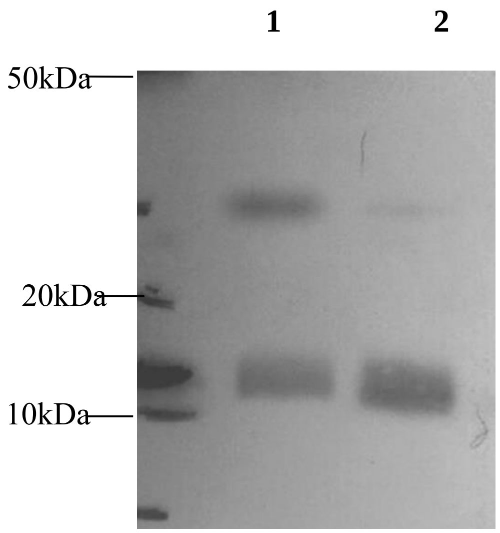

Supplementary Figure 2. α-syn heterocomplexes with tau and $A\beta_{1-42}$ in RBCs. Cell lysates obtained from RBCs of PD patients (line 1) and HC (line 2) were immunoprecipitated with an anti-α-syn antibody, and then immunoblotted with antibody to α-syn. One representative Western blot is presented.
